# The impact of COVID-19 restrictions on the presentation of self-injury: experience at a tertiary centre

**DOI:** 10.1192/bjo.2021.614

**Published:** 2021-06-18

**Authors:** Tobias Adams, A Arnaout, S Bickerton, L Li, AWN Reid, SH Leow

**Affiliations:** 1 Cambridge University Hospitals NHS Foundation Trust, University of Cambridge; 2 Cambridge University Hospitals NHS Foundation Trust

## Abstract

**Aims:**

The national lockdowns due to the COVID-19 pandemic, have had a considerable effect on mental health, with reduced access to social interaction through work and leisure activities, and increased barriers to community mental health services.

This study aims to evaluate how the presentation of patients with self-injury has changed during the first national U.K. lockdown, at a Plastic Surgery Tertiary Referral Centre in the East of England.

**Method:**

Retrospectively recorded data from 23 March 2020 to 31 December 2020, spanning the duration of the first two lockdowns in the UK, were compared to the same period in 2019. The demographics of patients, along with the nature, severity and outcomes of the self-injury were recorded and compared.

**Result:**

The number of patients referred for self-injury reduced by 22.9% during lockdown (2020: N = 42/109, 2019: 67/109)

The most common attendance route was via ambulance during lockdown (2020:40.5% 2019: 31.3%) whilst the most common attendance route being via the front door in 2019 (2019: 35.8%, 2020: 26.2%)

The number of new presentations with no prior history of self-injury was higher in lockdown 38.1%) compared to 2019 26.9%.

The lockdown cohort had a smaller proportion of patients presenting with complications (2020: 9.5% vs 2019: 17.9%), less readmitted (2020: 11.9% vs 2019: 23.9%). Similar re-attendance rate (2020: 40.3% vs 2019: 38.1%) and re-intervention (2020: 13.4% vs 2019: 14.3%).

A greater proportion in 2020 met the threshold for inpatient psychiatry input (2020: 52.4% vs 2019: 41.8%).

During the lockdown, a higher percentage of flexor tendon injuries involved multiple tendons (60.0% vs 52.2%). A higher percentage of extensor tendon injuries (14.3% vs 7.4%), and a greater proportion of these also involved multiple tendons (66.7% vs 40.0%). More self-injuries were complicated by fractures (7.1% vs 4.5%) and more required soft tissue reconstruction (11.9% vs 3.0%).

**Conclusion:**

Despite fewer patients presenting with self-injury during the 2020 lockdown, the injuries were more severe. Many of which had multiple structural injuries, and some with life-changing injuries, this is in line with our clinical observations.

During lockdown there was a higher proportion of first-time presentations without a history of self-injury and an increased need for inpatient psychiatry input. This may reflect the impact on mental health as a result of restricted social interactions.

These findings demonstrate the impact of lockdown on mental-health and may help inform medical services of potential changes in the presentations in future national social restrictions.

